# Regulating Critical Period Plasticity: Insight from the Visual System to Fear Circuitry for Therapeutic Interventions

**DOI:** 10.3389/fpsyt.2013.00146

**Published:** 2013-11-11

**Authors:** Elisa M. Nabel, Hirofumi Morishita

**Affiliations:** ^1^Graduate School of Biomedical Sciences, Icahn School of Medicine at Mount Sinai, New York, NY, USA; ^2^Department of Psychiatry, Icahn School of Medicine at Mount Sinai, New York, NY, USA; ^3^Department of Neuroscience, Icahn School of Medicine at Mount Sinai, New York, NY, USA; ^4^Department of Ophthalmology, Icahn School of Medicine at Mount Sinai, New York, NY, USA; ^5^Mindich Child Health and Development Institute, Icahn School of Medicine at Mount Sinai, New York, NY, USA; ^6^Friedman Brain Institute, Icahn School of Medicine at Mount Sinai, New York, NY, USA

**Keywords:** critical period, visual cortex plasticity, fear erasure, perineuronal nets, lynx1, HDAC inhibitors, reconsolidation update

## Abstract

Early temporary windows of heightened brain plasticity called critical periods developmentally sculpt neural circuits and contribute to adult behavior. Regulatory mechanisms of visual cortex development – the preeminent model of experience-dependent critical period plasticity-actively limit adult plasticity and have proved fruitful therapeutic targets to reopen plasticity and rewire faulty visual system connections later in life. Interestingly, these molecular mechanisms have been implicated in the regulation of plasticity in other functions beyond vision. Applying mechanistic understandings of critical period plasticity in the visual cortex to fear circuitry may provide a conceptual framework for developing novel therapeutic tools to mitigate aberrant fear responses in post traumatic stress disorder. In this review, we turn to the model of experience-dependent visual plasticity to provide novel insights for the mechanisms regulating plasticity in the fear system. Fear circuitry, particularly fear memory erasure, also undergoes age-related changes in experience-dependent plasticity. We consider the contributions of molecular brakes that halt visual critical period plasticity to circuitry underlying fear memory erasure. A major molecular brake in the visual cortex, perineuronal net formation, recently has been identified in the development of fear systems that are resilient to fear memory erasure. The roles of other molecular brakes, myelin-related Nogo receptor signaling and Lynx family proteins – endogenous inhibitors for nicotinic acetylcholine receptor, are explored in the context of fear memory plasticity. Such fear plasticity regulators, including epigenetic effects, provide promising targets for therapeutic interventions.

## Introduction

As the brain develops, particular regions undergo different critical periods of plasticity when their underlying circuits gain heighted sensitivity to experience (1, 2). Experience during these early temporal periods has a profound effect on the wiring of skills and behaviors, such as language, music playing, visual processing, and emotional processing. When the critical period for a region closes, the adaptations in its circuitry become fixed, locking in adjusted ways of processing and responding to stimuli and bringing plasticity into a latent state. This mechanism is normally a beneficial way to retain optimized behaviors without need for maintenance or renewal. However, in individuals exposed to inappropriate stimuli, adaptive changes that were helpful during this window of developmental plasticity may not be beneficial in the future and can lead to dysfunctional behavior. Understanding the mechanisms that open and close critical period development can inform interventional strategies that attempt to modify these pathways later in life. In this review, we turn to the visual cortex as a well-developed model of experience-dependent critical period plasticity to provide novel insights for the mechanisms regulating plasticity in the fear system.

## Critical Periods Across Brain Functions

### Critical period for visual cortex plasticity

**Figure 1:**
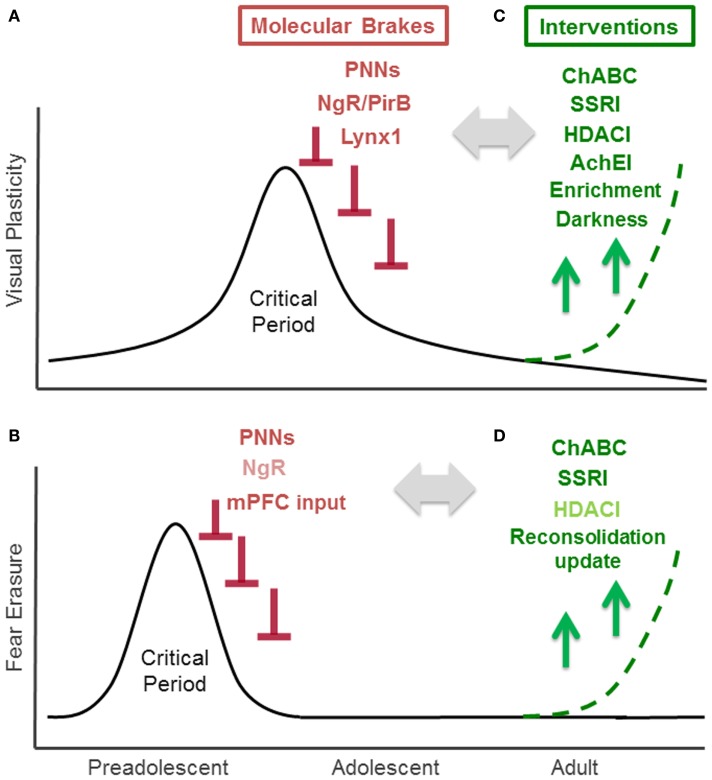
**Critical Period for experience-dependent plasticity in visual and fear system**. **(A)** Critical period of visual cortex plasticity: visual cortex development is the preeminent model for the study of critical period plasticity and its regulators. Visually depriving one eye by obstructing it early in life yields a life-long loss of visual acuity (amblyopia). Studies of mouse visual cortex development, which has a well-defined 2 weeks critical period that peaks at 1 month after birth, have identified several endogenous “molecular brakes” (colored in red) that close the critical period. These include perineuronal nets (PNNs), myelin-related Nogo receptor (NgR) and PirB, and a nicotinic brake Lynx1. **(B)** Critical period of fear erasure: a critical period for the fear system near adolescence is emerging through current research. In rodents, extinction can permanently erase fear memory during a preadolescent critical period around 17 days after birth, however, extinction at 24 days or later fails to bar fear memories from re-emerging. Increased PNNs in the amygdala and maturation of input from medial prefrontal cortex (mPFC) to amygdala contribute to the closure of this critical period. Nogo receptor (NgR: colored in light red) also limits fear extinction, but its contribution on fear erasure is not tested. **(C)** Interventions for enhancing visual cortex plasticity in the adult: counteracting molecular brakes through pharmacological (ChABC, SSRI, HDACI, AChEI: colored in green) and behavioral (environmental enrichment, dark exposure) approaches is a promising therapeutic strategic for recovery from amblyopia. **(D)** Interventions for fear erasure in the adult: juvenile-like plasticity can be reintroduced in adulthood through pharmacological treatment (ChABC, SSRI treatment) or reconsolidation update. HDACI administration (colored in light green) enhances extinction, but its effect on fear erasure has not been examined yet.

The visual system offers valuable insight through the study of critical period mechanisms. In humans and animals, visually depriving one eye by obstructing it early in life yields loss in visual acuity (amblyopia) by stimulating an anatomical remodeling within primary visual cortex (3). Importantly, such an effect of visual deprivation has not been seen in the adult, strongly suggesting the presence of a developmental critical period for visual experience-dependent plasticity in visual cortex. Due to a lack of sufficient brain plasticity in adulthood, untreated monocular deprivation during childhood results in life-long amblyopia, a condition affecting 2–5% of the human population (4). Indeed, recovery from deprivation amblyopia in adulthood is limited across species, from higher mammals (3), to rodents (5, 6), and requires therapeutic intervention. Over the last 10 years, the murine visual system, has emerged as a valuable model system for creating such intervention, having a well-defined, 2 week critical period that peaks 1 month after birth (Figure 1A). The predictability and duration of this temporal window is particularly useful for dissecting the molecular mechanisms of visual cortex plasticity through genetic manipulation and environmental intervention (7). Critical period mechanisms identified in rodent visual cortex have not only catalyzed multiple pharmacological and behavioral interventions that aid functional recovery in adults (8), but have also guided research uncovering molecular mechanisms of critical period plasticity in other brain regions, especially the auditory and fear systems (9–11).

### Critical period for fear memory erasure

Evidence from both animal and human studies suggest that the pathways underlying fear systems also undergo age-related changes in experience-dependent plasticity. Such age-related changes have been observed in both fear memory acquisition and extinction. Fear acquisition, measured through the ability to develop conditioned context-shock fear responses, does not emerge until 13–14 days (12). However, the mechanism governing the age-dependent change is little explored. Developmental differences in fear extinction, characterized by the ability to re-encode a previously encoded fear response, are observed in human studies and translate to rodent models, providing an effective animal model for exploring fear circuitry plasticity mechanisms. Fear extinction may be temporary, or it can lead to permanent fear memory erasure. The outcome of fear extinction is age-dependent. Fear extinction during a critical period in preadolescent mice (P13-17), leads to permanent fear erasure (13, 14). On the other hand, mice extinguished 24 days after birth or later exhibit a returned fear response (15) (Figure 1B). The juvenile form of fear extinction is also marked by an accelerated rate of change compared to that of adult mice (16), but the mechanistic relationship between the persistence and rate of fear extinction is unknown. Current evidence also suggests that erasure is specific to early temporal windows following a traumatic event (1–3 days later), as rodents undergoing later extinction training exhibit long-term hyper-vigilance rather than complete erasure (17). Understanding the mechanisms underlying these age-related changes in fear system plasticity may contribute to the better understanding of fear disorders, such as post traumatic stress disorder (PTSD), that are characterized by the re-experiencing of the traumatic events, hyper-vigilance, and persistent dysfunctional wiring of fear circuitry (18, 19).

### Parallels between visual and fear system plasticity

To what extent are visual and fear system critical period mechanisms parallel? Both the visual and fear systems maintain a developmentally limited ability to rewire connections that are no longer functionally appropriate. In the visual system, critical period (but not adulthood) monocular deprivation triggers a functional re-adaptation via visual cortex restructuring that reduces the input from the visually deprived eye. Early development fear extinction modifies behavior by erasing fear memories that are no longer appropriate, and differs from adult extinction that is temporary and slower. Thus, to discuss the role of molecular regulators in critical periods of plasticity, we consider them within their functional contexts and in functional parallels between systems.

To interrogate the extent to which the regulatory mechanisms of critical period plasticity in the visual system apply to the fear system, we conceptually consider “permanent fear erasure” analogously to “amblyopia,” both of which are only induced during critical period. Accordingly, we also consider “fear extinction procedure” in fear system and “monocular deprivation” in the visual system as the inducers of plasticity in each system. Because the juvenile form of fear extinction is also marked by accelerated changes in response compared to that of adult mice (16), we consider both the rate and persistence of fear memory extinction as the measures of fear plasticity. Although the following discussion focuses on this parallel nature of plasticity between visual and fear system development, it should be noted that additional mechanisms likely contribute to the age-related changes described, including fear memory erasure due to increased rates of neurogenesis during development (20).

## Molecular Brakes: Common Mechanisms for Critical Period Closure?

Recent studies using rodent visual cortex have identified multiple structural and functional molecular “brakes” that actively limit plasticity and close the critical period in the adult brain (8, 21). Structural brakes include PNNs (22), myelin-related inhibitory signaling mediated by Nogo receptor (23), and paired immunoglobulin-like receptor B expression (PirB) (24). Functional brakes, such as the nicotinic receptor binding protein Lynx1 act upon excitatory-inhibitory balance within local circuits (25). Importantly, lifting these brakes can induce critical period plasticity in adulthood and re-introduce ocular dominance remodeling following monocular deprivation. Here, we consider the potential roles of these major brakes explored in the visual system within fear circuitry.

### Perineuronal nets

Perineuronal nets are extracellular macromolecular aggregates associated with several subclasses of chondroitin sulfate proteoglycans (CSPGs) that surround neuronal cell bodies and proximal dendrites (26). In the visual system, PNNs inhibit experience-dependent plasticity observed during the critical period (Figure 1A). Further organization of CSPGs into PNNs coincides with the end of the critical period. Interestingly, PNN degradation with chondroitinase-ABC, an enzyme that degrades a key linkage glycoprotein and attacks CSPG side chains that allow the aggregates to form, restores experience-dependent plasticity in adult rats (22). Mice lacking a cartilage link protein that attenuates PNNs, Crtl1, consistently retain juvenile levels of plasticity in the adult visual cortex (27). Further, chondroitinase-ABC treatment coupled with reverse lid-suturing in adult rats – opening the sutured lid of the visually deprived eye while suturing the lid of the other, visually active eye – causes a complete recovery of ocular dominance to the originally deprived eye. This shift is accompanied by an increase in visual acuity and dendritic spine density (28). Although the underlying mechanisms by which PNNs halt plasticity remain elusive, a possible explanation for the protective action of PNNs is that they change the dynamics of local GABAergic inhibition. PNNs form primarily around parvalbumin (PV)-positive GABAergic interneurons, which are involved in the onset of critical period in the visual cortex. Recent studies further showed that the expression of PNNs is regulated by the cellular transfer of the homeodomain transcription factor Otx2 from the retina and Choroid-plexus to the visual cortex. This homeoprotein signals PV maturation in GABAergic interneurons and contributes to both the opening and closure of the critical period (29–32) (Figure 1A).

In the fear system, the PNNs in the amygdala recently have been shown to play a central role in modifying the plasticity of fear memories and may contribute to protection against fear memory lability during acquisition. The number of CSPG containing PNNs increases in the murine amygdala between days 16 and 23 – the time coinciding with preadolescence and the developmentally related functional switch from fear erasure to less effective fear extinction (10) (Figure 1B). A recent study removing amygdala PNNs with chondroitinase-ABC supports the role of PNNs as a plasticity brake that actively protects fear memories from erasure (10). When adult mice are injected with chondroitinase-ABC prior to fear conditioning and followed by extinction training 2–3 days later, fear extinction proceeds at a rate similar to that observed in juvenile mice, suggesting a greater response to extinction training (Figure 1B). Interestingly, chondroitinase-ABC was only effective when injected before fear conditioning; injection prior to extinction but after fear training was ineffective. Thus, the CPSG aggregation is likely involved in the initial fear memory encoding, and plays a role in maintaining the extinction-resistance of the fear memory rather than regulating the processes which occur during extinction. When the fear response was tested several weeks after the extinction training, the mice did not show a fear response to either the conditioned stimulus or the context of fear training, while control mice showed substantial renewal of the fear response (10). Thus formation of PNNs in fear systems may alter the function of local inhibitory circuits to promote the formation of extinction-resistant memory trace during fear conditioning, and degradation of these nets may allow for more inhibitory connectivity to protect against fear memory lability. In a post-mortem case control study, schizophrenic patients exhibited reduced numbers of PNNs in the lateral amygdala nuclei and entorhinal cortex (33). Further, oxidative stress, frequently observed in peripheral tissues and brains of schizophrenia patients, has been shown to delay the formation of PNNs (34, 35). The disruption of PNNs may play a central role in disease pathogenesis. It is currently difficult to discern whether the PNN is exerting an effect on fear acquisition or extinction because the downstream effectors of PNN disruption have not yet been identified. To better understand the role of the PNN in fear systems, further studies are needed to examine how PNNs are controlled and how they exert their effects.

### Myelin-related Nogo receptor signaling

In the visual cortex, experience-dependent plasticity is limited by myelin-related Nogo receptor signaling (23). The end of the visual critical period coincides with the maturation of intracortical myelin, which contains myelin-related inhibitory proteins such as NogoA, MAG, and OMgp, all of which bind to the neuronal Nogo receptor (36) (Figure 1A). Nogo receptor knock-out mice maintain normal levels of plasticity during the critical period, however the plasticity is maintained beyond critical period for up to 120 days postnatally (23). Additionally, PirB, a paired receptor with high affinity for Nogo, is also found to restrict ocular dominance plasticity in the visual cortex (24) (Figure 1A). The Nogo receptor was recently shown to be also involved in determining the rate of synaptic turnover in the adult cerebral cortex, as knock-out mice have increased levels of synaptic turnover (37). These myelin-related brakes may also regulate structural plasticity at the level of dendritic spine.

In a recent study, Nogo receptor knock-out mice were reported to show more pronounced fear extinction compared to wild type mice (37) (Figure 1B). At the dendritic spine level, fear extinction 3–4 days after fear conditioning involves spine growth on the same dendritic branches within 2 μm from the spines that were eliminated during conditioning in the cortex (38). Removal of a Nogo receptor brake may return high synaptic turnover which can result in stronger fear extinction learning (37). Whether or not Nogo receptor signaling contributes to fear erasure mechanisms in a fashion similar to PNNs is question that remains to be examined.

### Lynx family

While structural brakes can limit plasticity by altering local connectivity, functional brakes can also halt plasticity by altering the neurotransmission between connections that have been formed to facilitate plasticity. In the visual cortex, a newly discovered class of proteins, the Lynx family, has been recently identified as a class of functional brakes (25). Lynx1, an endogenous prototoxin similar to α-bungarotoxin in snake venom, acts by binding to the nicotinic acetylcholine receptor (nAChR) and limiting its activation (39). Increases in Lynx1 expression coincide with closure of the critical period in the adult mouse visual cortex. Further, removal of this molecular brake during adulthood re-induces a plastic state by acutely resetting local excitatory-inhibitory circuit balance. Lynx1 expression in adults suppresses functional plasticity into a latent state, as removal of this brake allows the critical period remains open until nAChR signaling is actively blocked (25) (Figure 1A). The adult Lynx1 knock-out mice that received amblyopic long-term visual deprivation during critical period showed spontaneously recovery of visual acuity to normal levels simply by reopening the closed eye (25). While a permissive role for ACh has long been appreciated during the critical period (40), it has remained a mystery why visual cortex plasticity is severely restricted in adulthood even in the presence of massive cholinergic innervation from the basal forebrain. Lynx1 provides a molecular basis for maintaining stability in the presence of ACh.

The Lynx family may also have developmental roles in fear system. Lynx1 and closed related Lynx2 are both expressed in the amygdala and change its expression levels across development in both rodent and human (41). Both Lynx1 and Lynx2 knock-out mice express an amplified response to cue fear conditioning, but demonstrate normal contextual fear conditioning in adulthood (42, 43). As the juvenile cue response is normally stronger than that of the adult (44), Lynx family proteins may dampen cue-conditioned fear learning from adolescence to adulthood. Considering its age-related changes in expression, the Lynx family may also have a role on extinction and critical period of fear memory erasure. Indeed, nicotine administration during extinction training over the course of 6 days after fear conditioning is reported to enhance extinction (45), however, the direct role of Lynx family proteins in fear memory erasure remain to be tested.

## Therapeutic Strategies Based on Critical Period Mechanisms

The ability to remove brakes on critical periods provides the opportunity to reopen windows of plasticity in order to remodel, or re-develop, the adult brain by re-introducing juvenile-like plasticity. Small molecules targeting these brakes carry potential clinical relevance for both neurological and psychiatric disease, including PTSD. It is well-established that administering d-cycloserine (DCS), a partial NMDA agonist, facilitates extinction, and prevents the recovery of fear memories in rats, mice, and humans both before and after extinction training (46–49). Here we discuss the possibility of additionally using three well-established drug classes administered in humans – selective serotonergic reuptake inhibitors (SSRIs), acetylcholinesterase inhibitors (AChEIs), and histone deacetylase (HDAC) inhibitors – to target both structural and functional plasticity brakes based on data from both animal and human studies (Figure 1C). Finally, we also consider behavioral interventions such as reconsolidation update, which may mimic or trigger similar effects.

### Serotonergic reuptake inhibitors

In animal studies, chronic SSRI treatment reintroduces juvenile-like plasticity to the adult visual cortex. Administration of fluoxetine, the first-line antidepressant SSRI, has been demonstrated to restore critical period plasticity in the adult rat brain (50). Chronic fluoxetine treatment in adult rats not only induced visual cortex plasticity after monocular deprivation, but also improved visual acuity in amblyopic animals (Figure 1C). Specifically, rats were treated with fluoxetine for 4 weeks underwent monocular deprivation on day 21 of treatment. Increased BDNF levels in the visual cortex were accompanied by reduced GABAergic inhibition, likely restoring excitatory-inhibitory balance by reopening visual critical period plasticity.

Plasticity reactivation by chronic SSRI treatment has recently been examined in the fear system. Strikingly, fear erasure is facilitated in adult mice by SSRI treatment and resembles fear erasure in non-treated juvenile mice (Figure 1D) (51). Chronic fluoxetine treatment for 3 weeks prior to and throughout the duration of fear conditioning does not influence the encoding of a conditioned fear response, but rather causes faster extinction and permanent erasure compared to mice that undergo extinction training without prior SSRI treatment. This effect is mediated through BDNF pathways: BDNF expression was exaggerated in the basolateral amygdala by SSRI treatment, and the effect of SSRI on fear erasure was absent in mice heterozygous for BDNF allele. Interestingly, SSRI treatment starting after the fear acquisition was sufficient to induce faster extinction and permanent erasure, a salient difference compared to fear erasure induced by PNN disruption, which was only effective when treated before and not after fear conditioning. Fluoxetine treated mice had similar number of PNN-positive neurons compared to control but had a reduced percentage of PNN expressing parvalbumin interneurons in the basolateral amygdala. Together, these data suggest that fluoxetine treatment selectively shifts parvalbumin interneurons toward an immature state, inducing critical period-like plasticity in local inhibitory neurons in the basolateral amygdala. The effects of chronic fluoxetine treatment (4–5 weeks) were also seen in CA1 of the hippocampus, where SSRIs have been shown to return mature granule cells to an electrophysiologically immature state, with reduced synaptic facilitation in the mossy fibers (52). Loss of hippocampal synaptic proteins has been associated with a PTSD-like syndrome in mice, and is counteracted with SSRI treatment for 4 weeks of chronic fluoxetine treatment prior to fear conditioning (53).

### Acetylcholinesterase inhibitors

Another class of pharmaceutical agents shown to induce recovery of visual function in the adulthood is the acetylcholinesterase inhibitor (AchEI). In the visual system, AchEI injection can restore vision in adult wild type mice with amblyopia, a disorder in which the eye, though structurally normal, has impaired vision due to poor functional connectivity to the visual cortex (25) (Figure 1C). By increasing cholinergic tone, AChEI may counteract the nicotinic functional brake Lynx1 on critical period plasticity.

AchEI treatment has not been directly examined in the context of fear extinction and memory liability, but nicotine dosing immediately before training has been reported to enhance extinction training (45). As AChEI can rapidly activate BDNF receptor TrkB in hippocampus, AChEI may have similar effect on fear memory liability to SSRI (54). However, further studies are clearly needed to better elucidate the effects that AchEI have on fear system plasticity.

### HDAC inhibitors

In the visual system, experience-dependent modifications of histone acetylation are developmentally down-regulated, implicating epigenetic mechanisms in the regulation of critical period plasticity (55). Administration of the HDAC inhibitor trichostatin A in adult mice reactivates visual cortex plasticity (55) and chronic administration of two separate HDAC inhibitors, valproic acid and sodium butyrate, to amblyopic adult mice undergoing long-term monocular deprivation induces recovery of visual acuity following reverse lid-suturing (56) (Figure 1C). Although the effect of HDAC inhibitors is intriguing, the changes in genetic expression profiles that HDAC inhibitors produce, and subsequent downstream effects of visual cortical plasticity, are still unknown. One possibility is that HDAC inhibitors are regulating gene expression of molecular brakes, such as myelin-related molecules, as histone modifications are involved in oligodendrocyte precursor cell differentiation during development. Some potential gene targets include transcription factors required for myelination, such as SOX10 and Krox-20. Administration of an HDAC inhibitor during myelination onset, which coincides with the fall of the visual cortex critical period, prevents oligodendrocyte precursor cell maturation (57–59). However, further analyses are required to unravel the effectors of the epigenetic treatment on visual plasticity and to confirm that effects are specific enough to plasticity brakes to be of clinical benefit.

In the fear system, systemic, as well as direct, application of HDAC inhibitors into the hippocampus or medial prefrontal cortex (mPFC) prior to fear conditioning and prior to extinction enhances extinction learning (60–64) (Figure 1D). Oral administration of an HDAC inhibitor enhances extinction learning in response to weak extinction protocols that are ineffective when administered on their own, and direct application of HDAC inhibitors to the hippocampus and mPFC increases c-fos expression. Future studies are necessary to determine whether HDAC inhibitors also promote permanent erasure of fear memory. Research comparing adult histone acetylation activity to juvenile activity will also inform whether acetylation has developmental specificity and mechanistic contributions to the critical period for fear lability.

### Behavioral interventions

#### Visual system

Environmental enrichment (65) and dark exposure (66) have been reported as effective behavioral interventions for recovery from amblyopia in adult rats (Figure 1C). These interventions may reset excitatory – inhibitory balance, thus re-introducing juvenile-like plasticity in the adult brain (8). The effects of behavioral interventions on recovery from adult amblyopia have also been examined in humans. Perceptual learning, involving extensive practice on a challenging discrimination between simple visual stimuli (67–69), and action videogames, which requires the allocation of spatial attention, detection, and localization of low contrast, fast moving targets, emerge as tools for visual acuity improvements in adult amblyopia (70, 71). These interventions may induce plasticity by either lifting molecular brakes through invasive interventions or by exploiting endogenous permissive factors such as neuromodulators (21).

#### Fear system

A newly proposed behavioral intervention for treating PTSD, “reconsolidation update,” involves targeting traumatic memories as an individual reconsolidates it because the memory is rendered labile after it has been recalled (Figure 1D). Extinguishing a fear response during the window of reconsolidation prevents the reinstatement of a fear response to a stimulus at a later time point in both humans and in rodents (72–74). It is notable that the mechanisms of this process are localized to the amygdala and exclude the mPFC (75, 76). The mPFC is thought to be involved in reactivation of emotional states associated with past experiences, which may account for the lower rate of PTSD among war veterans with selective mPFC damage (77). The mPFC is also uninvolved in juvenile fear systems (Figure 1B) (78). Extinction during reconsolidation involves the amygdala in both juvenile mice and humans, and the mPFC is uninvolved in adolescent rat extinction (15, 79). During reconsolidation, events in the amygdala may open a temporal window of experience-dependent plasticity that allows a fear memory to be degraded by new experiences. However, it should be also noted that facilitation of extinction may also happen in addition to reconsolidation update (80). The molecular mechanisms of fear erasure by reconsolidation update are only now beginning to be explored. Recent work suggests that phosphorylation of the AMPA receptor subunit GluA1 regulates this process (72). There is also increasing evidence that reconsolidation is accompanied by epigenetic changes, pointing to a potential effect of epigenetic regulation on the cellular alterations underlying experience-dependent plasticity (81–85). Whether or not reconsolidation update limits the expression of molecular brakes remains open to future investigation. Combining reconsolidation update and pharmacological interventions may be a fruitful future direction.

## Conclusion

In this review, we considered the potential contributions of “molecular brakes” identified in visual system development, the major model of critical period plasticity, to the development of fear system connections. Striking similarities between the molecular mechanisms underlying the development of these two brain regions, as well as therapeutic approaches to their dysfunction, indicate that new mechanisms identified in the visual critical period can provide both novel insights and a conceptual framework for exploring novel therapeutic approaches to aberrant fear responses in PTSD patients. Future studies examining the contributions of molecular brakes, such as myelin-related nogo receptor signaling and the Lynx family, as well as their epigenetic regulators in the context of the fear system development, may shed light on new targets for therapeutic intervention. The interactions between multiple brakes have yet to be connected – not only those mentioned in this review, but also well-established age-related mechanisms, such as hippocampal neurogenesis. Future research manipulating critical period mechanisms for clinical use will require further elucidation of systems in which these molecular brakes are involved. The mechanism of re-closure after the therapeutic reopening the critical period is another important area of investigation. Finally, better comprehension of the scope of these regulators to realize both their therapeutic potential as well as the undesired consequences of increased brain plasticity is needed.

## Conflict of Interest Statement

The authors declare that the research was conducted in the absence of any commercial or financial relationships that could be construed as a potential conflict of interest.
